# Spatiotemporal Expression and Substrate Specificity Analysis of the Cucumber *SWEET* Gene Family

**DOI:** 10.3389/fpls.2017.01855

**Published:** 2017-10-27

**Authors:** Yaxin Li, Sheng Feng, Si Ma, Xiaolei Sui, Zhenxian Zhang

**Affiliations:** Beijing Key Laboratory of Growth and Developmental Regulation for Protected Vegetable Crops, College of Horticulture, China Agricultural University, Beijing, China

**Keywords:** cucumber, phylogeny, spatiotemporal expression, subcellular localization, substrate specificity, sugar, SWEET transporters

## Abstract

The functions of SWEET (Sugar Will Eventually be Exported Transporter) proteins have been studied in a number of crops, but little is known about their roles in cucumber (*Cucumis sativus* L.), a model plant for studying stachyose metabolism and phloem function. Here, we identified 17 cucumber *SWEET* genes (*CsSWEETs*), located on chromosomes 1–6, and classified them into four clades. Two genes from each clade were selected for spatiotemporal expression, subcellular localization, and substrate specificity analyses. Clade I and II proteins were all hexose transporters and targeted to the plasma membrane, while clade III proteins also localized to the plasma membrane, but used sucrose as a substrate. Clade IV SWEET proteins were localized to the tonoplast, and used hexose as a substrate. The eight tested *CsSWEET* genes were most highly expressed in flower, which represents a large sink in plants. However, each gene also showed specific expression patterns: three of the eight tested genes were highly expressed in mature leaves, two in roots, two in fruit, two in stems, and one was detected in all tested organs. The likely biological roles of each are discussed based on the above results.

## Introduction

Sugar Will Eventually be Exported Transporters (SWEET) proteins are membrane localized proteins with seven transmembrane domains (TMs) that have been identified as sugar transporters (STPs) by co-expression studies with the high-sensitivity fluorescence resonance energy transfer (FRET) glucose/sucrose sensor in human HEK293T cells (Chen et al., 2010; Xuan et al., 2013; Eom et al., 2015). Unlike sucrose transporter (SUT), STP (symporter) or vacuolar glucose transporter 1 (VGT1) (antiporter), SWEET proteins are energy-independent and their action is driven by a substrate concentration gradient across the membrane (Chen et al., 2015a). SWEET proteins are widespread in land plants, but have also been found in algae (*Chlamydomonas reinhardtii*), nematodes (*Caenorhabditis elegans*), and in mammals (Chen et al., 2010). In angiosperms, the number of reported *SWEET* genes varies from 8 to 52, depending on the species, with 8 in the earliest diverging angiosperm, *Amborella trichopoda*, 17 in *Arabidopsis thaliana*, 17 in grapevine (*Vitis vinifera*), 21 in rice (*Oryza sativa*), 23 in sorghum (*Sorghum bicolor*), 29 in tomato (*Solanum lycopersicum*), 35 in potato (*S. tuberosum*), 47 in eucalyptus (*Eucalyptus grandis*), and 52 in soybean (*Glycine max*) (Chen et al., 2010; Eom et al., 2015; Feng et al., 2015; Manck-Götzenberger and Requena, 2016; Mizuno et al., 2016).

Chen et al. (2010) first classified SWEET transporters into four clades, and identified differences in their subcellular localization, substrates and functions. SWEET proteins from clades I and II preferentially transport hexoses, mainly glucose, and they have been reported to be localized to the plasma membrane, with the exception of SWEET2 from *A. thaliana. AtSWEET5* (*VEX1*) is expressed in mature, hydrated and germinating pollen and is found specifically in the vegetative cell of pollen grains, consistent with a role in supplying the generative cell with sugars (Engel, 2005; Eom et al., 2015). In rice, the clade II protein, OsSWEET5, is a galactose transporter and is expressed in anthers. *OsSWEET5*-overexpressing plants were shown to display a growth retardation phenotype and precocious senescence at the seedling stage (Zhou et al., 2014). The clade II protein AtSWEET8 (RPG1) is preferentially expressed in inflorescences, and may transport sugar to the tapetum and the microsporocyte/microspores, which function as a subunit for the synthesis of primexine precursors and callose during the meiosis and tetrad stage. Knocking out the expression of *AtSWEET8* resulted in reduced primexine deposition and pollen degradation (Guan et al., 2008; Sun et al., 2013). ZmSWEET4c and OsSWEET4 also appeared to be responsible for transferring hexoses across the basal endosperm transfer layer (BETL) to sustain development of the large starch-storing endosperm of cereal grains and contribute to sink strength (Sosso et al., 2015).

SWEET proteins from clade III are sucrose transporters and are targeted to the plasma membrane (Chen et al., 2012). Genes in this clade have various functions, including exporting sucrose from the phloem parenchyma cells (PPC) prior to phloem loading in source tissues, embryo development, nectar secretion and pollen nutrition by the tapetum, which are mostly symplast-isolation spaces in sink tissues (Chen et al., 2012, 2015b; Sun et al., 2013; Lin et al., 2014). SWEET9, also referred to NEC1 in petunia (Wittich, 2000), has been identified as being nectar-specific and to play essential roles in nectar secretion in *A. thaliana*, *Brassica rapa*, and *Nicotiana attenuata*. Specifically, the protein acts to release sucrose from the nectary parenchyma cells to the apoplast, whereupon it is hydrolyzed by cell wall invertases to produce glucose and fructose (Lin et al., 2014). AtSWEET11 and -12, also from clade III, release sucrose from leaf PPCs to the apoplast, after which it is trafficked by SUT transporters into the sieve element-companion cell complex (Chen et al., 2012; Eom et al., 2015). The *atsweet11;12* double mutant was reported to accumulate starch in its leaves and the cumulative exudation of [^14^C]-derived assimilates from ^14^CO_2_ fed cut petioles was reduced (Chen et al., 2012), providing strong evidence for the corresponding proteins functioning in phloem loading. The rice ortholog, OsSWEET11 (Os8N3/Xa13), is also shown to be a sucrose transporter (Chen et al., 2012). In a later study, it was found that AtSWEET proteins also contribute to assimilate transfer from the maternal seed coat to the developing embryo (Chen et al., 2015b). AtSWEET11, -12, and -15 are expressed in the seed coat and endosperm, and the corresponding triple knockout mutant showed a severe delay in embryo development, as well as a wrinkled seed phenotype at maturity due to a low starch and lipid content and a smaller embryo (Chen et al., 2015b; Eom et al., 2015). AtSWEET15 (SAG29) functions in remobilization of carbohydrates during senescence (Seo et al., 2011), and AtSWEET13 (RPG2) is shown to partially alleviate the loss of AtSWEET8 function at the late reproductive stage in the *atsweet8* mutant, suggesting some level of redundancy. In addition, the *atsweet8;13* double mutant shows a high degree of sterility at the late reproductive stage, with small siliques and few seeds (Sun et al., 2013).

SWEET proteins from Clade IV are vacuolar hexose transporters, and may play roles in balancing intracellular hexose homeostasis. As an example, AtSWEET17 functions as a fructose-specific uniporter in the root and leaf tonoplast, thereby playing a key role in facilitating bi-directional fructose transport across the tonoplast of the roots and leaves to maintain cytosolic fructose homeostasis (Chardon et al., 2013; Guo et al., 2014).

Additionally, SWEET protein function has been associated with pathogen infection and it was reported that the expression of various *SWEET* genes can be induced by biotrophic bacteria or fungi, to release sugar to the apoplast, thereby promoting pathogen growth (Chen et al., 2010; Chen, 2014). This phenomenon has been confirmed in many crop plants (Yuan et al., 2009; Antony et al., 2010; Chen et al., 2010; Liu et al., 2011; Chen, 2014; Chong et al., 2014; Manck-Götzenberger and Requena, 2016).

Although considerable progress has been made in the study of SWEET proteins from many plant species, to date little is known about their expression or function in a cucurbit, even though members of the Cucurbitaceae are model plants for studying stachyose metabolism, as well as phloem function. Recently, the cucumber (*Cucumis sativus* L.) genome sequence was published (Huang et al., 2009), providing a basis for studying the cucumber *SWEET* gene family. Hu et al. (2017) identified 17 SWEET transporters in cucumber by bioinformatic analysis. In this current study, phylogenetic analyses, yeast uptake assays, quantitative real-time PCR (qRT-PCR), and green fluorescent protein (GFP) fusion protein localization were used to determine the classification, substrate specificity, spatiotemporal expression profiles, and subcellular localization of the cucumber SWEET genes/proteins. The resulting data are used to infer their likely biological functions, thereby providing a theoretical basis for further research of SWEET proteins in cucurbit species.

## Materials and Methods

### Plant Material and Bacteria/Yeast Strains

Cucumber (*C. sativus* L. cv. Xintaimici) plants were grown under glasshouse conditions with 28/18°C (day/night) temperature and 12/12 h (light/dark) photoperiod in Beijing, China. Tissue including root, stem, young leaf (sink leaf in apical growing point), mature leaf (functional leaf), flowers, and fruit were collected from 3-month-old plants and used for qRT-PCR analyses. The DH5a *Escherichia coli* strain was used for cloning and *Saccharomyces cerevisiae* strains EBY.VW4000 and SUSY7/ura were used for heterologous protein expression.

### Identifying Members of the Cucumber *SWEET* Gene Family and Phylogenetic Analyses

*CsSWEET* genes were identified via BLASTN searches in the National Center for Biotechnology Information (NCBI^1^) database and the Cucurbit Genomics Database (CuGenDB^2^). The resulting 17 cucumber putative *SWEET* genes were named *CsSWEET1-17*, with the numbering based on their homologous genes in *A. thaliana* (Chen et al., 2010). The letter corresponds to the location on chromosome and Hu et al. (2017).

Phylogenetic analyses were conducted using ClustalW in Mega 5.0 software and an unrooted phylogenetic tree of the CsSWEET, AtSWEET, and SlSWEET families were constructed using Mega 5.0 software according to Chen et al. (2010). The evolutionary history was inferred using the neighbor joining method with 1,000 replicates. The evolutionary distances were computed using the Poisson correction distance model and are given as the number of amino acid substitutions per site.

### Gene Structure and Transmembrane Domain Analysis

Information on the SWEET genes was acquired from NCBI, including locus tags, gene symbols, accession numbers, the chromosomal position and the MtN3 saliva family (PFAM database code PF03083^3^) analysis. Structure of each *CsSWEET* gene was analyzed using NCBI. The TMs were analyzed using TMHMM^4^.

### Heterologous Expression of CsSWEET in Yeast

The *CsSWEET1*, *CsSWEET2*, *CsSWEET5a*, *CsSWEET7b*, *CsSWEET10*, *CsSWEET12c*, *CsSWEET17a*, and *CsSWEET17c* open reading frames (ORFs) were amplified by PCR and cloned into the *S. cerevisiae*/*E. coli* shuttle vector pDR196 (Fan and Zhang, 2009). PCR primers listed in Supplementary Table S3.

The recombinant vectors or the empty pDR196 vector (control) were separately transferred into the hexose uptake-deficient yeast strain EBY.VW4000 and sucrose uptake-deficient yeast strain SUSY7/ura, as previously described (Cheng et al., 2015). The transformed cells of the hexose uptake-deficient strain were grown on SD (synthetic deficient) medium supplemented with 2% maltose (glucose for sucrose uptake-deficient strain) and auxotrophic requirements. Serial dilutions (×10, ×10^2^, × 10^3^) of yeast cell suspensions (CsSWEET1, CsSWEET2, CsSWEET5a, CsSWEET7b, CsSWEET17a, and CsSWEET17c) were dropped on solid SD media containing either 2% maltose (control) or 2% glucose/fructose/galactose/mannose/xylose/arabinose plus respective auxotrophic requirements (Cheng et al., 2015). Serial dilutions (×10, ×10^2^, ×10^3^) of CsSWEET10, CsSWEET12c were plated on SD media containing either 2% glucose (control) or 2% sucrose plus respective auxotrophic requirements. Growth was documented by pictures after 2–5 days growth at 30°C.

### Expression Profiles of Cucumber *SWEET* Genes

Gene expression analyses were performed using qRT-PCR analysis with the SYBR green detection protocol (TaKaRa, Japan) and the ABI 7500 system (Bio-Rad, United States). Total RNA was extracted from specified tissues (root, stem, young leaf, mature leaf, male flower, female flower, fruit on the day of anthesis and fruit on the ninth day after anthesis) using RNAprep pure Plant Kit (Tiangen, Beijing, China), and then reverse-transcribed using FastQuant RT Kit (with gDNase) (Tiangen, Beijing, China). The cDNA samples were then used as a template for qRT-PCR analysis. Primers are listed in Supplementary Table S3.

### Subcellular Localization of CsSWEET Proteins

The *CsSWEET1*, *CsSWEET7b*, *CsSWEET12c*, and *CsSWEET17a* ORFs without stop codons were amplified by RT-PCR using gene specific primers and the amplicons were cloned into the pCAMBIA super 1300 vector to generate C-terminal fusions with the GFP reporter. Primers are listed in Supplementary Table S3. Transient expression of the CsSWEET1-GFP, CsSWEET7b-GFP, CsSWEET12c-GFP, and CsSWEET17a-GFP fusion proteins in *A. thaliana* protoplasts and onion epidermal cells were performed according to Ma et al. (2008) and Fan and Zhang (2009), respectively. The empty vector expressing untargeted GFP was used as a control. GFP fluorescence was visualized using an Olympus Confocal Laser Scanning Microscope (FV1000, Japan).

## Results

### Identification and Phylogenetic Analysis of the Cucumber SWEET Family

**FIGURE 1:**
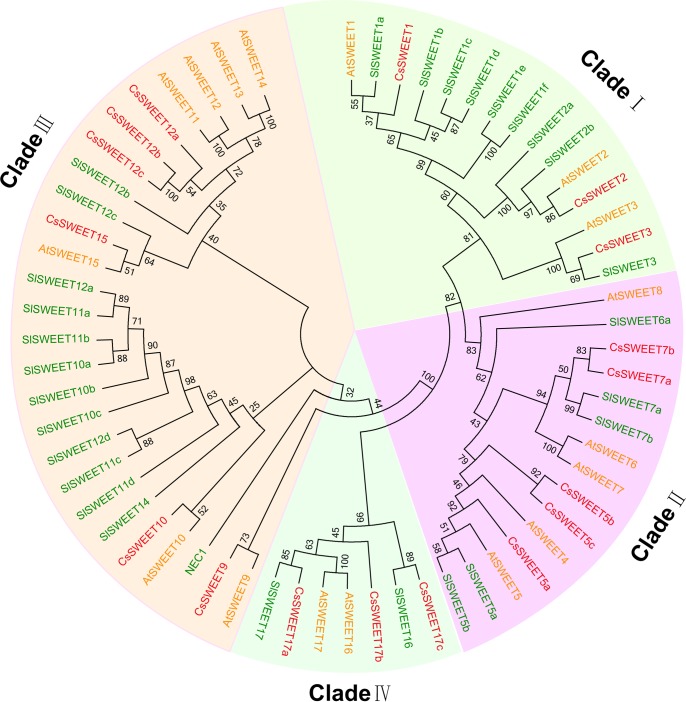
Phylogenetic tree of cucumber (*Cucumis sativus*), *Arabidopsis thaliana*, and tomato (*Solanum lycopersicum*) SWEET proteins. AtSWEET is *A. thaliana* (yellow), CsSWEET is *Cucumis sativus* (red), and SlSWEET is *Solanum lycopersicum* (green). The evolutionary history was inferred using the neighbor joining method with 1,000 replicates. The evolutionary distances were computed using the Poisson correction distance model and are in the units of the number of amino acid substitutions per site. Evolutionary analyses were conducted using MEGA5.0 software. Accessions are listed in Supplementary Table S2.

Seventeen candidate cucumber *SWEET* genes (*CsSWEET*) were identified and named according to their homologs in *A. thaliana* (Chen et al., 2010) (**Table 1**). Sequences of the *CsSWEETs* were based on BLAST searches against the NCBI (NCBI^5^) database and Cucurbit Genomics Database (CuGenDB^6^). A phylogenetic tree based on the 17 CsSWEET protein sequences, together with 17 AtSWEET sequences and 29 SlSWEET sequences was constructed using MEGA5.0 software. The cucumber sequences clustered into the four clades that have been reported in the *A. thaliana* and *O. sativa* families (Chen et al., 2010) (**Figure 1**). Clade I contained CsSWEET1-3, besides, three AtSWEETs and nine SlSWEETs; Clade II contained CsSWEET5a-c, 7a-b, besides, five AtSWEETs and five SlSWEETs; Clade III contained CsSWEET9-10, 12a-c, 15, besides, seven AtSWEETs and thirteen SlSWEETs; Clade IV contained CsSWEET17a-c, besides, two AtSWEETs and two SlSWEETs. Sequence identity comparisons among the four CsSWEET protein subclasses were 28–77%, and the average identity was 42%. CsSWEET12b and CsSWEET12c showed the highest homology (77%), CsSWEET5b and CsSWEET5c, CsSWEET7a, and CsSWEET7b had 64 and 62% homology, respectively (Supplementary Table S1).

**Table 1:** Cucucmber SWEETs information.

Gene name	Locus tag	Gene symbol	Accession		AA	Chr	Gene	Exon	TMs	MtN3	
CsSWEET1	Csa6M343690.1	LOC101217779	XM_004150675.2	XP_004150723.1	252	6	3856	6	7	6–94	131–215
CsSWEET2	Csa4M622870.1	LOC101220605	XM_004146030.2	XP_004146078.2	233	4	2787	6	7	16–102	135–218
CsSWEET3	Csa5M139600.1	LOC101215611	XM_011656324.1	XP_011654626.1	259	5	2751	6	7	25–111	145–231
CsSWEET5a	Csa1M046010.1	LOC101222361	XM_004152504.2	XP_004152552.1	238	1	1546	6	7	10–95	133–218
CsSWEET5b	Csa3M836410.1	LOC101217357	XM_004141139.2	XP_004141187.1	236	3	1910	6	6	19–95	131–212
CsSWEET5c	Csa3M836420.1	LOC101217589	XM_011653972.1	XP_011652274.1	247	3	4626	6	7	9–94	134–214
CsSWEET7a	Csa4M054810.1	LOC101207440	XM_004148937.2	XP_004148985.1	261	4	3226	5	7	10–98	132–218
CsSWEET7b	Csa5M148830.1	LOC101206775	XM_004143811.2	XP_004143859.1	265	5	2582	5	7	10–98	134–218
CsSWEET9	Csa2M083730.1	LOC101218764	XM_004138930.2	XP_004138978.2	262	2	3902	6	7	11–97	134–206
CsSWEET10	Csa1M001300.1	LOC101210005	XM_004137984.2	XP_004138032.1	292	1	1876	5	7	10–96	131–211
CsSWEET12a	Csa1M001290.1	LOC101221611	XM_004138202.2	XP_004138250.1	275	1	1523	5	7	13–100	135–219
CsSWEET12b	Csa1M051710.1	LOC101212917	XM_004153453.1	XP_004153501.1	295	1	2238	6	7	13–99	134–219
CsSWEET12c	Csa1M051720.1	LOC101221861	XM_004145098.2	XP_004145146.2	291	1	1824	6	7	12–98	133–218
CsSWEET15	Csa6M092510.1	LOC101220116	XM_004140499.2	XP_004140547.1	277	6	3024	6	7	12–95	134–218
CsSWEET17a	Csa1M569280.1	LOC101203449	XM_004135553.2	XP_004135601.1	295	1	6428	6	6	7–94	129–214
CsSWEET17b	Csa2M031160.1	LOC105434717	XM_011651808.1	XP_011650110.1	228	2	2571	6	7	12–98	133–218
CsSWEET17c	Csa3M159450.1	LOC101206107	XM_004134172.2	XP_004134220.1	244	3	3820	6	7	6–91	129–213


*Data in this table are from NCBI database. AA, amino acid, Chr, chromosome, TMs, transmembrane domains, MtN3, MtN3 saliva family (PFAM database code PF03083; http://pfam.xfam.org).*

### Gene Structure and Transmembrane Domain Analysis

To analyze the structural characteristics of the *CsSWEET* genes, we examined their conserved regions, and aligned the putative protein sequences. Their exon numbers varied among the genes, *CsSWEET7a, 7b*, *CsSWEET10*, and *CsSWEET12a* containing five exons, and the other gene family members all containing six exons (**Table 1**).

SWEET proteins have seven TMs in eukaryotes, as well as two MtN3/saliva domains. They contain a basic 3-TM unit and a functional transporter containing at least four TMs (tetramer) (Xuan et al., 2013). There are 80–90 amino acids in the two cucumber MtN3/saliva domains, and they are present at almost the same positions in all the proteins, as shown in **Table 1**.

**FIGURE 2:**
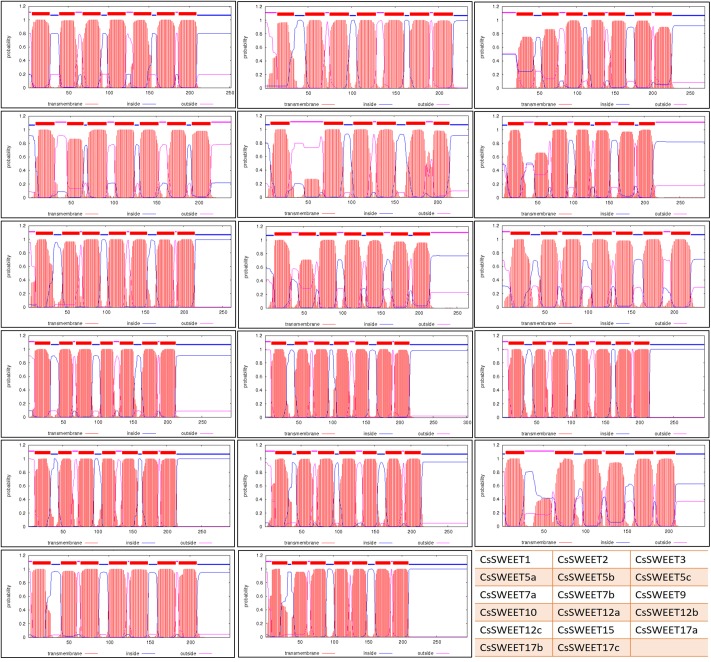
The transmembrance domains of CsSWEET proteins. The position of N- and C-terminal domains of the protein are indicated by blue or pink lines. Table on the right bottom shows the location of each protein in this figure. The website used for predictions was TMHMM.

To confirm the presence of the TMs, the amino acid sequences were submitted to the TMHMM Server v.2.0^7^. The results confirmed that all, except 2 of the 17 CsSWEETs have seven TMs, CsSWEET5b and CsSWEET17a, which have six TMs (**Figure 2**). The N-terminal of CsSWEET1, 2, 3, 7a, 9, 10, 12a, 12b, 12c, 15, 17b, 17c is outside of the membrane, while it is on the inside for CsSWEET5a, 5b, 5c, 7b, 17a (**Figure 2**).

### Heterologous Expression and Substrate Specificity Analysis in Yeast

**FIGURE 3:**
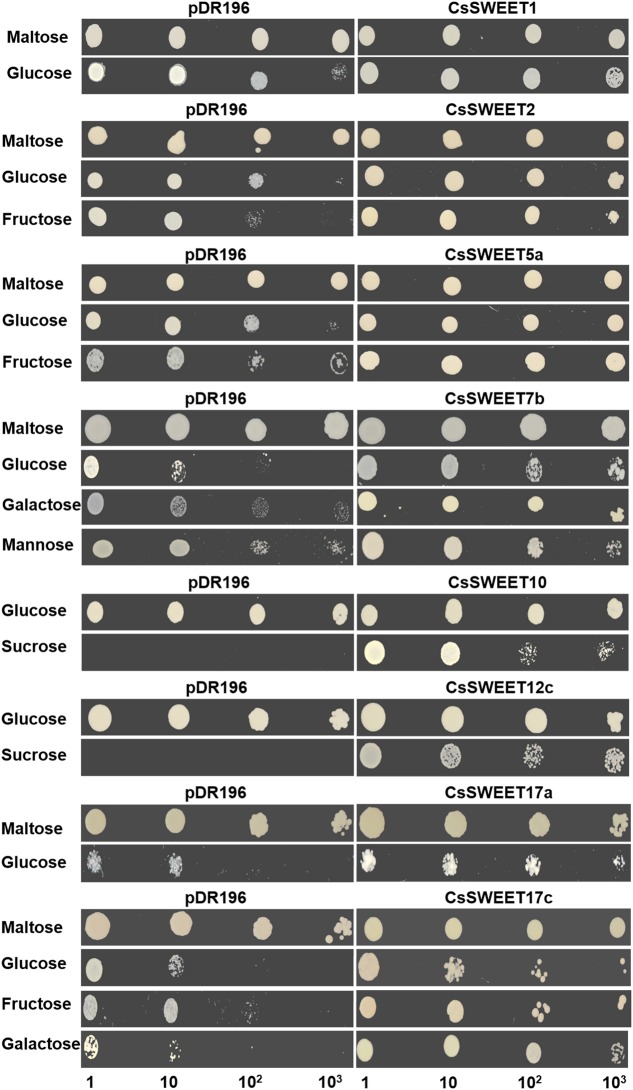
Heterologous expression of eight *CsSWEET* genes in yeast. Yeast strains with recombinant vectors or the empty pDR196 vector (as a negative control) were grown on SD (synthetic deficient)-ura medium supplemented with 2% maltose or different sugars (listed above) as the sole carbon source for 5 days. Medium with maltose as sole carbon source is a positive control for strain EBY.WV4000, while with glucose as sole carbon source is a positive control for strain SUSY7/ura. Yeast cell suspensions were diluted (×10, ×10^2^, ×10^3^) for serial dilutions assay. The other results of the experiment are in Supplementary Figure S2.

In order to identify the substrates being transported by the CsSWEET proteins, the hexose uptake-deficient yeast mutant EBY.VW4000 and the sucrose uptake-deficient yeast mutant SUSY7/ura3 (Riesmeier et al., 1992; Wieczorke et al., 1999) were used. We isolated six full-length cDNA clones, which encoded putative hexose transporters and two full-length cDNA clones, which encoded putative sucrose transporters, from cucumber male flowers. We then determined that CsSWEET1 and CsSWEET2 from clade I allowed glucose uptake, or glucose and fructose uptake, in the yeast EBY.VW4000 mutant (**Figure 3**), but did not show detectable complementation of uptake of other hexoses (Supplementary Figure S2). From clade II, CsSWEET5a supported uptake of glucose and fructose, while CsSWEET7b conferred glucose, galactose and mannose uptake (**Figure 3**), but not of other hexoses. From clade III, both CsSWEET10 and CsSWEET12c conferred sucrose uptake in the yeast SUSY7/ura3 mutant but did not allow hexose uptake in the EBY.VW4000 mutant (**Figure 3** and Supplementary Figure S2). Finally, from clade IV, CsSWEET17a allowed glucose uptake in the yeast EBY.VW4000 mutant, while CsSWEET17c enabled glucose, galactose and fructose uptake (**Figure 3**), but not of other hexoses (**Figure 3** and Supplementary Figure S2).

In conclusion, CsSWEET proteins from clades I, II, and IV transport hexoses, such as fructose, galactose, mannose, and particularly glucose. CsSWEET proteins from clade III transport sucrose, rather than hexoses, which is similar to results from *A. thaliana* and rice (**Figure 3**).

### Spatiotemporal Expression Analysis of the *CsSWEET* Genes

**FIGURE 4:**
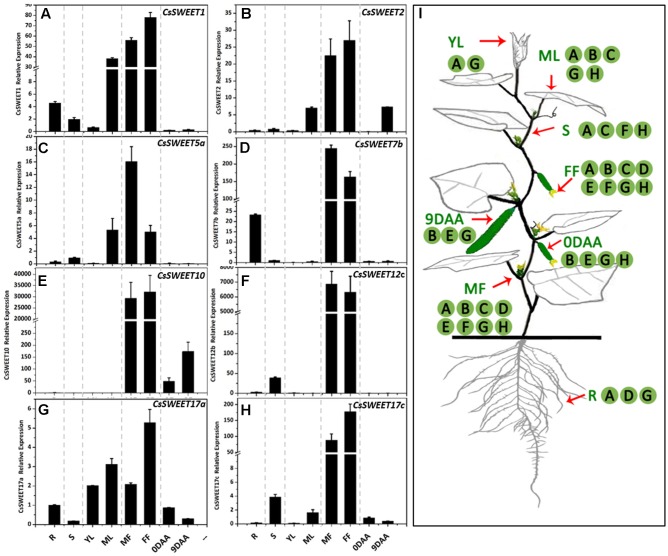
The expression patterns of eight *CsSWEET* genes. **(A–H)** Relative expression of *CsSWEETs*. R, root; S, stem; YL, young leaf; ML, mature leaf; MF, male flower; FF, female flower; 0DAA, ovary/fruit on the day of anthesis, 9DAA, fruit on the ninth day after anthesis. **(I)** Schematic model of the areas of *CsSWEET* gene expression in cucumber. Letters in **(I)** match the expression pattern in **(A–H)**. Error bars represent the SE for three technical replicates of three biological replicates.

In order to determine the spatiotemporal expression pattern of each *CsSWEET* gene, the transcriptional profile in different cucumber organs was analyzed using qRT-PCR. Samples of fruit at the marketable fruit maturation stage 9 DAA (days after anthesis) (Hu et al., 2011) and other organs, including a source organ (mature leaf), sink organs (root, male flower, female flower, fruit, and young leaf) and a transporting organ (stem), were isolated. The eight analyzed *CsSWEET* genes from the four clades were expressed in almost all organs (**Figure 4I**), and at very high levels in male and female flowers, especially in the case of *CsSWEET7b*, *-10*, *-12c*, and *-17c*, where the expression was approximately 10–100 times greater than in other organs (**Figure 4**). But beyond that, the genes from different clades showed distinct expression patterns. In clade I, *CsSWEET1* and *CsSWEET2* were highly expressed in mature leaves, with *CsSWEET1* also showing high expression in roots and *CsSWEET2* in 9 DAA fruit, while they both showed low expression in the other organs (**Figures 4A,B**). In clade II, *CsSWEET5a* showed high expression in mature leaves, and low expression in roots and stems. *CsSWEET7b* was mainly expressed in roots, with little expression in stems, source leaves and fruit (**Figures 4C,D**). In clade III, *CsSWEET10* was specifically expressed in fruit, while *CsSWEET12c* was expressed only in the stems and flowers (**Figures 4E,F**). In clade IV, *CsSWEET17a* was expressed in almost all organs, but with a high level in young leaves and mature leaves, while *CsSWEET17c* was highly expressed in stems besides flowers, and less in mature leaves and fruits (**Figures 4G,H**).

### Subcellular Localization of CsSWEET Proteins

**FIGURE 5:**
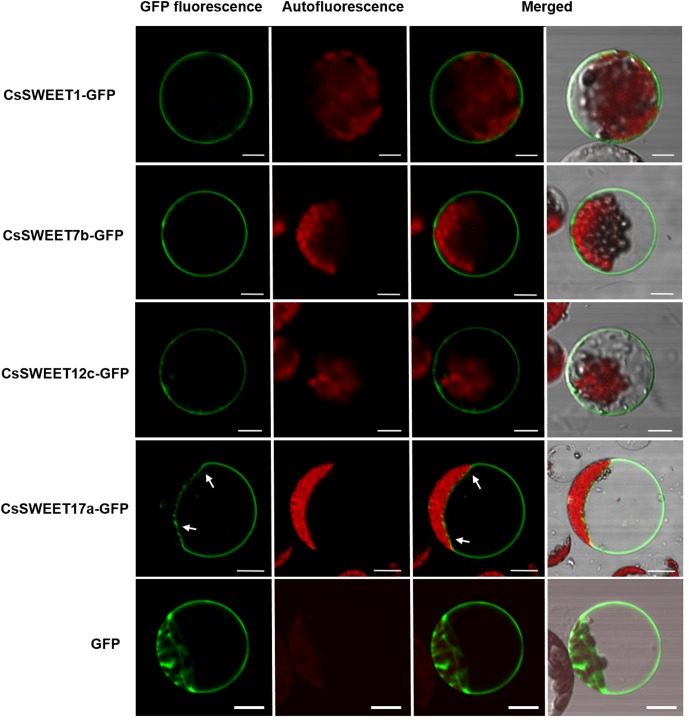
The subcellular localization of CsSWEET1, -7b, -12c, -17a green fluorescent protein (GFP) fusions in *A. thaliana* protoplasts. The white arrows indicate the tonoplast. Scale bars = 10 μm.

Previous studies have reported that different AtSWEET proteins have different subcellular localizations (Chen et al., 2015a; Eom et al., 2015), which in turn is suggestive of distinct functions. Here, the subcellular localization of the cucumber CsSWEET proteins was determined by expressing CsSWEETs-GFP fusion proteins in *A. thaliana* protoplasts. We found that CsSWEET1-GFP from clade I, CsSWEET7b-GFP from clade II and CsSWEET12c-GFP from clade III were targeted to the plasma membrane, while CsSWEET17a-GFP from clade IV localized to the tonoplast (**Figure 5**). These results were also verified in chloroplast-free onion (*Allium cepa*) epidermal cells (Supplementary Figure S1), suggesting that proteins from clades I, II, and III are plasma membrane proteins that may be involved in transmembrane transportation between cells, while clade IV proteins are tonoplast transporters, and may be involved in transportation and accumulation inside cells and in balancing intracellular hexose homeostasis.

## Discussion

In the earlier work (Hu et al., 2017), a genome-wide characterization of SWEET genes was conducted in cucumber (*Cucumis sativus* L.) including a phylogenetic tree of the SWEET genes in cucumber, *A. thaliana* and rice, analysis of cis-elements in promoter regions, chromosome distribution, gene structure and an analysis of transcript levels which provided a basic understanding of the *CsSWEET* genes. In their study, 17 *CsSWEETs* were identified which were not evenly distributed over the seven cucumber chromosomes. Besides that, *cis*-elements were identified in the promoter regions: nine types involved in phytohormone responses and eight types involved in stress responses (Hu et al., 2017). SWEETs have been reported as playing vital roles in embryo and seed development, phloem loading, nectary secretion, and other important life processes (Chen et al., 2012, 2015a; Lin et al., 2014; Sosso et al., 2015). These functions mostly depend on their subcellular localization and transport substrates. Based on the earlier work, phylogenetic analyses, yeast uptake assays, qRT-PCR and GFP fusion protein localization were used to elucidate the function of SWEET genes in cucumber in this study.

### *CsSWEET* Genes Fall into Four Phylogenetic Clades

In this study, 17 putative cucumber *SWEET* genes were identified, classified into four clades (I–IV), and named according to their homologs in *A. thaliana* and earlier work (Hu et al., 2017). The overall sequence identity of all cucumber SWEET proteins is 42%, while the equivalent identity for the *A. thaliana* SWEET proteins is 43%, and the degree of identity among all SWEET proteins shown in the phylogenetic tree was 33.03% (**Figure 1**). We identified six cucumber *SWEET* genes in clade III, five genes in clade II, and three genes in each of clades I and IV. This is similar to the numbers in *A. thaliana* and other plant species. In clade III, *A. thaliana* has 7 genes, tomato (*Solanum lycopersicum*) has 13, potato (*Solanum tuberosum*) has 15, and *Medicago truncatula* has 10 (Feng et al., 2015; Manck-Götzenberger and Requena, 2016). This relatively high number may be related to the important role of sucrose in long distance transport. In contrast there are only three clade IV genes in cucumber and potato, one in *M. truncatula* and rice, and two in *A. thaliana* and tomato.

### Substrate Specificity of CsSWEET Proteins

We determined that CsSWEET proteins from clades I and II could mediate uptake of hexose, especially glucose, in the yeast EBY.VW4000 mutant, which has also been reported for their homologs from *A. thaliana* (Chen et al., 2010, 2015a). In our study, we detected some differences between the uptake preferences of the different CsSWEET proteins: CsSWEET2 allowed fructose uptake, CsSWEET5a allowed the uptake of fructose and glucose, while CsSWEET7b conferred the uptake of glucose, and to a degree mannose, as well as galactose (**Figure 3**). In other species, less research has been done on substrate specificity. ZmSWEET4c and OsSWEET4 function as glucose and fructose transporters, VvSWEET4 encodes a glucose transporter and OsSWEET5 encodes a galactose transporter in yeast (Guo et al., 2014; Zhou et al., 2014; Sosso et al., 2015). Clade III was reported to be a sucrose-specific clade in *A. thaliana* (Chen et al., 2012), CsSWEET10 and CsSWEET 12c, also belong to clade III and mediated the uptake of sucrose, but not hexoses in the EBY.VW4000 mutant. In clade IV, the *A. thaliana* protein AtSWEET17 was found to be a fructose-specific transporter (Guo et al., 2014), while CsSWEET17a rescued glucose uptake and CsSWEET17c rescued glucose, galactose, and fructose uptake in the yeast mutant.

We therefore conclude that there may exist some differences in the functions of the SWEET proteins among species, and that the SWEET proteins generally have a broader substrate not only uptake glucose but other hexoses. Recently, it has been found that SWEETs have the functions of transporting plant hormone, such as gibberellin (Kanno et al., 2016).

### The Probable Biological Functions of the CsSWEET Proteins

Hu et al. (2017) predicted the following subcellular localizations of CsSWEETs by WoLF PSORT (a localization prediction tool); CsSWEET1 in the chloroplast/vacuole, CsSWEET12c and CsSWEET17a in the chloroplast. In our study, using GFP fusions, we discovered that CsSWEET1, CsSWEET7b, and CsSWEET12c are plasma membrane proteins, while CsSWEET17a is a vacuole membrane protein (**Figure 5**). These results, obtained through experimental tests differ from those of Hu et al. (2017) predicted by bioinformatics software/tools.

In this study, all of the eight tested *CsSWEET* genes were most highly expressed in male and female flowers, which are strong carbon sinks. SWEET proteins have been reported as having roles in nectar secretion and also tapetum and pollen formation (Guan et al., 2008; Sun et al., 2013; Lin et al., 2014), and in flowers, a lack of sugar availability may lead to flower abortion (Lebon et al., 2008; Chong et al., 2014). *CsSWEET1* and *CsSWEET7b* showed high expression in roots and flowers, and may be involved in the uptake and release of carbohydrates in these organs, or alternatively function in an interaction between roots and soil microorganisms. They may also play important roles in sugar partitioning during flower and root growth development (**Figures 4**, **5**). This is similar to AtSWEET4, which is a hexose transporter that mediates sugar transport to axial sinks (Liu et al., 2016). ZmSWEET4c and OsSWEET4 also appeared to be responsible for transferring hexoses across the BETL to sustain development of the large starch-storing endosperm of cereal grains and contribute to sink strength (Sosso et al., 2015).

Most studies of SWEET proteins in sink organs have focused on flowers, seeds or roots (Guan et al., 2008; Chardon et al., 2013; Sun et al., 2013; Guo et al., 2014; Chen et al., 2015b). The roles in fleshy sink organs, such as fruits, is a new and important area of research. In cucumber fruits, phloem unloading follows an extensive apoplastic pathway and needs transporter participation (Hu et al., 2011). In this study, *CsSWEET2* and *CsSWEET10* showed high levels of expression in fruit and their substrates were found to be hexoses and sucrose, respectively. Accordingly, sucrose may be released into the phloem parenchymal cells by CsSWEET10 transmembrane transport and glucose and fructose derived from sucrose degradation may be released into the phloem parenchymal cells by CsSWEET2. Their high expression levels in fruit is consistent with a possible role in apoplastic sugar unloading and fruit development.

In contrast, CsSWEET17a was found to be a vacuolar transporter (**Figure 5**) and showed 47.1% similarity with CsSWEET17c. They are both in clade IV and show 41.55 and 51.8% similarity with AtSWEET17, respectively, which is known to be a vacuolar transporter (Chardon et al., 2013; Guo et al., 2014). They were expressed in almost all the sampled organs as they are tonoplast proteins. We speculate that these genes, from clade IV, play a role in facilitating hexose transport across the tonoplast and in balancing intracellular hexose homeostasis.

## Conclusion

Based on their spatiotemporal expression patterns and subcellular localization, we propose that the CsSWEET proteins from clades I, II, and III may participate in the distribution of carbohydrate between organs through the phloem, because these corresponding proteins are localized in the plasma membrane. CsSWEET proteins from clade IV may be involved in hexose transport and accumulation inside cells and in balancing intracellular hexose homeostasis as they are targeted to the tonoplast. In future studies, the biological functions of the individual CsSWEET proteins will be investigated through histochemical localization and reverse genetics approaches.

## Author Contributions

ZZ, XS, and YL designed the research; YL, SF, and SM performed the research; YL and SF analyzed the data; YL, ZZ, and XS wrote the paper.

## Conflict of Interest Statement

The authors declare that the research was conducted in the absence of any commercial or financial relationships that could be construed as a potential conflict of interest.

## Footnotes

1 http://www.ncbi.nlm.nih.gov/

2 http://www.icugi.org/cgi-bin/ICuGI/index.cgi

3 http://pfam.xfam.org

4 http://www.cbs.dtu.dk/services/TMHMM

5 http://www.ncbi.nlm.nih.gov/

6 http://cucurbitgenomics.org/

7 http://www.cbs.dtu.dk/services/TMHMM
