# Supplementary material for: Spatiotemporal Expression and Substrate Specificity Analysis of the Cucumber SWEET Gene Family
Source: Front Plant Sci. 2017 Oct 27;8:1855. doi: 10.3389/fpls.2017.01855 (PMC5664084; doi:10.3389/fpls.2017.01855)
Supplement: Supplementary file 1 [file Table_1.PDF]

Table S1. Pairwise comparison of amino acid identity (%) of the 17 CsSWEET proteins.

	CsSWEET1	CsSWEET2	CsSWEET3	CsSWEET5a	CsSWEET5b	CsSWEET5c	CsSWEET7a	CsSWEET7b	CsSWEET9	CsSWEET10	CsSWEET12a	CsSWEET12b	CsSWEET12c	CsSWEET15	CsSWEET17a	CsSWEET17b	CsSWEET17c
**CsSWEET1**	100																
**CsSWEET2**	43.1	100															
**CsSWEET3**	39.3	36.2	100														
**CsSWEET5a**	39.6	34.6	33.2	100													
**CsSWEET5b**	40.8	34.5	34.3	53.4	100												
**CsSWEET5c**	36.9	32.0	32.2	53.8	63.6	100											
**CsSWEET7a**	36.9	36.4	32.2	53.4	45.8	45.2	100										
**CsSWEET7b**	37.5	35.1	33.5	50.8	48.7	44.3	61.8	100									
**CsSWEET9**	36.8	35.0	32.9	30.9	36.7	33.3	33.8	36.3	100								
**CsSWEET10**	33.2	31.6	29.0	31.8	35.9	29.1	33.1	34.5	56.2	100							
**CsSWEET12a**	34.5	35.2	31.1	28.2	31.0	25.4	28.9	27.7	52.3	46.4	100						
**CsSWEET12b**	35.5	35.4	29.7	31.3	36.8	31.4	33.6	30.6	51.9	47.5	54.8	100					
**CsSWEET12c**	32.8	35.7	29.6	31.5	36.9	29.8	32.8	30.8	52.3	47.8	54.5	76.6	100				
**CsSWEET15**	33.6	33.0	31.5	27.7	30.9	29.2	29.1	27.7	48.5	45.8	44.0	50.4	52.6	100			
**CsSWEET17a**	37.2	32.0	33.3	38.6	42.9	35.6	38.6	35.3	41.8	37.2	33.3	36.2	34.6	32.2	100		
**CsSWEET17b**	35.7	30.9	32.4	33.2	37.9	35.4	31.7	31.3	38.3	31.9	31.9	35.6	33.9	32.3	45.0	100	
**CsSWEET17c**	43.5	32.4	32.9	37.4	39.5	38.2	34.7	36.0	39.2	35.7	35.4	32.5	31.1	31.6	45.5	47.1	100
