# Supplementary material for: Spatiotemporal Expression and Substrate Specificity Analysis of the Cucumber SWEET Gene Family
Source: Front Plant Sci. 2017 Oct 27;8:1855. doi: 10.3389/fpls.2017.01855 (PMC5664084; doi:10.3389/fpls.2017.01855)
Supplement: Supplementary file 2 [file Table_2.PDF]

Table S2. Naming of SWEET genes from *Arabidopsis thaliana* and *Solanum lycopersicum* corresponding to *gene ID.*

Name	Gene ID	Name	Gene ID
AtSWEET1	AT1G21460	SlSWEET2a	Solyc02g071520
AtSWEET2	AT3G14770	SlSWEET2b	Solyc07g062120
AtSWEET3	AT5G53190	SlSWEET3	Solyc03g007360
AtSWEET4	AT3G28007	SlSWEET5a	Solyc03g114200
AtSWEET5	AT5G62850	SlSWEET5b	Solyc06g071400
AtSWEET6	AT1G66770	SlSWEET6a	Solyc02g086920
AtSWEET7	AT4G10850	SlSWEET7a	Solyc08g082770
AtSWEET8	AT5G40260	SlSWEET7b	Solyc12g055870
AtSWEET9	AT2G39060	SlNEC1	Solyc09g074530
AtSWEET10	AT5G50790	SlSWEET10a	Solyc03g097580
AtSWEET11	AT3G48740	SlSWEET10b	Solyc03g097600
AtSWEET12	AT5G23660	SlSWEET10c	Solyc03g097610
AtSWEET13	AT5G50800	SlSWEET11a	Solyc03g097870
AtSWEET14	AT4G25010	SlSWEET11b	Solyc03g097570
AtSWEET15	AT5G13170	SlSWEET11c	Solyc06g072620
AtSWEET16	AT3G16690	SlSWEET11d	Solyc06g072640
AtSWEET17	AT4G15920	SlSWEET12a	Solyc03g097590
SlSWEET1a	Solyc04g064610	SlSWEET12b	Solyc03g097620
SlSWEET1b	Solyc04g064620	SlSWEET12c	Solyc05g024260
SlSWEET1c	Solyc04g064630	SlSWEET12d	Solyc06g072630
SlSWEET1d	Solyc04g064640	SlSWEET14	Solyc03g097560
SlSWEET1e	Solyc06g060590	SlSWEET16	Solyc01g099880
SlSWEET1f	Solyc06g060580	SlSWEET17	Solyc01g099870
