# Supplementary material for: Spatiotemporal Expression and Substrate Specificity Analysis of the Cucumber SWEET Gene Family
Source: Front Plant Sci. 2017 Oct 27;8:1855. doi: 10.3389/fpls.2017.01855 (PMC5664084; doi:10.3389/fpls.2017.01855)
Supplement: Supplementary file 3 [file Table_3.PDF]

Table S3. Primers used in this study.

Primers	Description	Sequence (5’- -3’)
SWEET1F	Open reading frame clone	AACTGCAGATGGATATTCCGCACTTCTTGTTC
SWEET1R	Open reading frame clone	CCGCTCGAGTTAAACTTGATCATCACGGTCCACT
SWEET2F	Open reading frame clone	AACTGCAGATGGTTCTTCTCGGTTCCAT
SWEET2R	Open reading frame clone	CCGCTCGAGCTACGCATAGGATACAATTAGAGGT
SWEET5aF	Open reading frame clone	AACTGCAGATGGTGAATACAGAGACAGCAAGA_
SWEET5aR	Open reading frame clone	CCGCTCGAGTCTACACATTATCTGTCATTTGCAC_
SWEET7bF	Open reading frame clone	AACTGCAGATGGTTTCTCCAGATGCC_
SWEET7bR	Open reading frame clone	CCGCTCGAGTCAGGCCTTCCGGGT_
SWEET10F	Open reading frame clone	AACTGCAGATGGCCATCAGTCCCCAAA
SWEET10R	Open reading frame clone	CCGCTCGAGTTAGTTAGTTATGATGTCTTGGTCTTT
SWEET12cF	Open reading frame clone	AACTGCAGATGGCTTTGTCCTTCAATAC
SWEET12cR	Open reading frame clone	CCGCTCGAGTTAAACATGATTAAGTTGATCTTT
SWEET17aF	Open reading frame clone	AACTGCAGATGGCCCTTAGTTTTATGAAT_
SWEET17aR	Open reading frame clone	CCGCTCGAGTCAAGCCTCACATTTGACC_
SWEET17cF	Open reading frame clone	AACTGCAGATGGCTTTATTCGATACTCATC_
SWEET17cR	Open reading frame clone	CCGCTCGAGTTAAACATGATTAAGGTGATCTGTA_
TubulinF	Quantitative RT-PCR	GTTCTTATCAATGCTGGTGGTG
TubulinR	Quantitative RT-PCR	TTTACTCACAGTCCCTTGGTCTC
SWEET1F	Quantitative RT-PCR	AACTGCCTTATTTGCCTATGGTATG
SWEET1R	Quantitative RT-PCR	GCGAACATTGATACGAGAGAAGC
SWEET2F	Quantitative RT-PCR	AACTGCCTTATTTGCCTATGGTATG
SWEET2R	Quantitative RT-PCR	GCGAACATTGATACGAGAGAAGC
SWEET5aF	Quantitative RT-PCR	ACGCTATTCTCAAGTTCGATCCTAA
SWEET5aR	Quantitative RT-PCR	CATTATCTGTCATTTGCACCTCTGG
SWEET7bF	Quantitative RT-PCR	ACGCCACCTTTTACAAATCAACTC
SWEET7bR	Quantitative RT-PCR	CCGTCGTGTCAGAAATAGGGG
SWEET10F	Quantitative RT-PCR	GATGCTGGCTTTGACTCTCG
SWEET10R	Quantitative RT-PCR	CAAAAGGCATATACTCCACGCTC
SWEET12cF	Quantitative RT-PCR	ATAGCCATAGCAAAGGAAATGAAGC
SWEET12cR	Quantitative RT-PCR	TGACTTCCAAGGTTTTGTGGTTATG
SWEET17aF	Quantitative RT-PCR	GTTTTCCTGATTTACGCACCTTC
SWEET17aR	Quantitative RT-PCR	CAATACGCTTTTCCCCTTCC
SWEET17cF	Quantitative RT-PCR	ACTGTCAATGGCGTTGGTGTT
SWEET17cR	Quantitative RT-PCR	CCTTCTTTTGTTTGGGAGCG
SWEET1F	Translational fusion to GFP	AACTGCAGATGGATATTCCGCACTTCTTGTTC
SWEET1R	Translational fusion to GFP	GGACTAGTAACTTGATCATCACGGTCCACT
SWEET7bF	Translational fusion to GFP	AACTGCAGATGGTTTCTCCAGATGCC
SWEET7bR	Translational fusion to GFP	GGACTAGTGGCCTTCCGGGT
SWEET12cF	Translational fusion to GFP	AACTGCAGATGGCTTTATTCGATACTCATC
SWEET12cR	Translational fusion to GFP	GGACTAGTAACATGATTAAGGTGATCTGTA
SWEET17aF	Translational fusion to GFP	AACTGCAGATGGCGGAGCTCAGCTTCT
SWEET17aR	Translational fusion to GFP	GGACTAGTGGGTTGTGGATGTGGAGAA
