# Supplementary material for: Spatiotemporal Expression and Substrate Specificity Analysis of the Cucumber SWEET Gene Family
Source: Front Plant Sci. 2017 Oct 27;8:1855. doi: 10.3389/fpls.2017.01855 (PMC5664084; doi:10.3389/fpls.2017.01855)
Supplement: Supplementary file 4 [file Image_1.pdf]

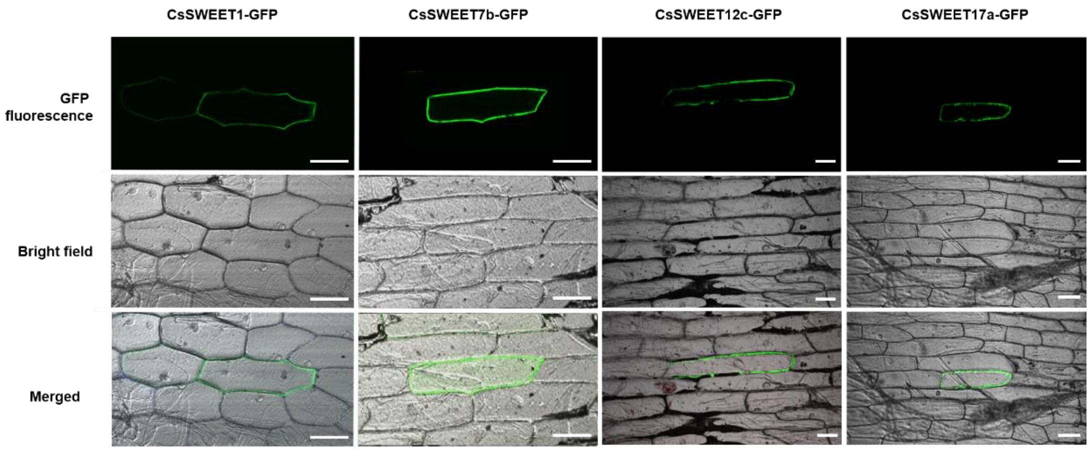

Fig. S1. The subcellular location of CsSWEET-green fluorescent protein (GFP) fusions in onion epidermal cells. Scale bars=50μm.
