# Supplementary material for: Spatiotemporal Expression and Substrate Specificity Analysis of the Cucumber SWEET Gene Family
Source: Front Plant Sci. 2017 Oct 27;8:1855. doi: 10.3389/fpls.2017.01855 (PMC5664084; doi:10.3389/fpls.2017.01855)
Supplement: Supplementary file 5 [file Image_2.pdf]

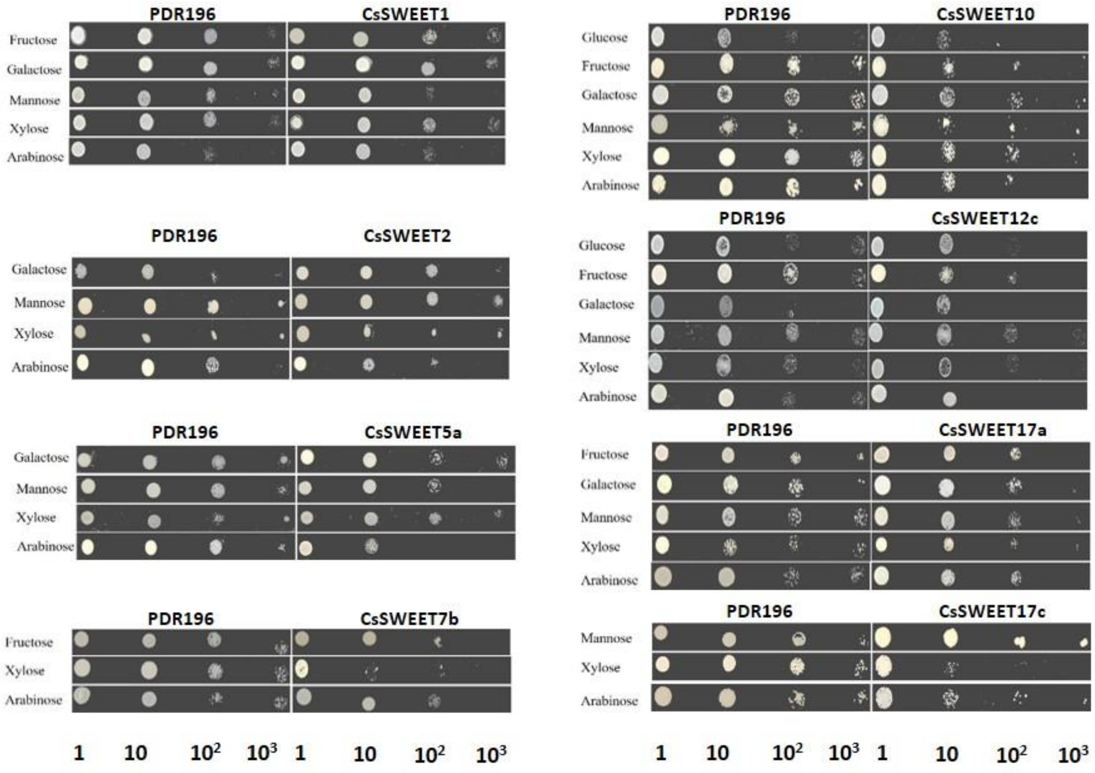

Fig. S2. Heterologous expression of eight *CsSWEET* genes in hexose uptake-deficient yeast strain EBY.VW4000. No obviously uptake were observed in recombinant vectors or the empty pDR196 vector (as a negative control) when grew on SD (synthetic deficient)-ura medium supplemented with 2% sugar (listed above) as the sole carbon.
